# Therapeutic Implications of TGFβ in Cancer Treatment: A Systematic Review

**DOI:** 10.3390/cancers13030379

**Published:** 2021-01-20

**Authors:** Verónica Gómez-Gil

**Affiliations:** Department of Biomedical Sciences (Area of Pharmacology), School of Medicine and Health Sciences, University of Alcalá, 28805 Alcalá de Henares, Madrid, Spain; email@vgomezgil.com or veronica.gomezg@uah.es

**Keywords:** TGFβ, cancer, drug target, antisense oligonucleotides, epithelial to mesenchymal transition, metastasis, miRNA, siRNA, SMAD, small molecule inhibitor

## Abstract

**Simple Summary:**

While the importance of transforming growth factor β (TGFβ) in cancer development and progression has long been recognized, a successful therapy targeting this cytokine has not been developed yet. The difficulty in blocking the tumor-promoting activity of this factor while maintaining the tumor suppressor effects can compromise the expected outcomes. This systematic review summarizes and discusses the different strategies being tested to regulate TGFβ expression in cancer treatment, as well as their associated side effects.

**Abstract:**

Transforming growth factor β (TGFβ) is a pleiotropic cytokine that participates in a wide range of biological functions. The alterations in the expression levels of this factor, or the deregulation of its signaling cascade, can lead to different pathologies, including cancer. A great variety of therapeutic strategies targeting TGFβ, or the members included in its signaling pathway, are currently being researched in cancer treatment. However, the dual role of TGFβ, as a tumor suppressor or a tumor-promoter, together with its crosstalk with other signaling pathways, has hampered the development of safe and effective treatments aimed at halting the cancer progression. This systematic literature review aims to provide insight into the different approaches available to regulate TGFβ and/or the molecules involved in its synthesis, activation, or signaling, as a cancer treatment. The therapeutic strategies most commonly investigated include antisense oligonucleotides, which prevent TGFβ synthesis, to molecules that block the interaction between TGFβ and its signaling receptors, together with inhibitors of the TGFβ signaling cascade-effectors. The effectiveness and possible complications of the different potential therapies available are also discussed.

## 1. Introduction

Transforming growth factor β (TGFβ) plays a relevant role in many different processes, such as cell migration and invasion, the inhibition of epithelial, hematopoietic, and immune cell growth [1], the epithelial-to-mesenchymal (EMT) transition or the extracellular matrix remodeling [2]. In line with the influence of TGFβ on these and other key cell/tissue mechanisms, the deregulation of its signaling cascade or an imbalance in its effects can lead to developmental defects and a wide variety of pathologies [3,4]. It is chronically overexpressed in diverse pathological states, such as fibrosis, inflammation, asthma, neointimal hyperplasia, rheumatoid arthritis, or multiple sclerosis [5]. TGFβ is significantly affected by aging. Thus, the association between TGFβ signaling and aging-associated disorders, such as Alzheimer’s disease, muscle atrophy, or impaired healing, has also been evidenced [4,6].

One of the most prevalent and severe diseases in which TGFβ comes into play is cancer. The role of TGFβ in cancer remains controversial, due to the opposing effects it can exert depending on the tumor stage. In healthy tissue and early-stage tumors, TGFβ limits cell proliferation and induces apoptosis, thus acting as a tumor suppressor [7]. Later on, the inactivation of its signaling cascade disappears, and cancer cells become able to escape these inhibitory effects. Then, the loss of response of tumor cells to TGFβ produces an increase in the secretion of this factor, which can act in an independent manner by promoting cell proliferation and invasion that will lead up to tumor progression [8]. Thereby, hyperactivation of the TGFβ signaling cascade is observed in established tumors [9]. TGFβ signaling cascade alterations, such as mutations affecting SMAD proteins or the blocking of their phosphorylation process by the inhibitory SMADs, SMAD6 and SMAD7, have been proved to be related to tumor progression [10]. In addition, mutations driving the inactivation of different members of the TGFβ signaling pathway were found in different types of cancer. Mostly, TβRII (TGFβ Type II receptor) is affected in colon carcinoma, breast, lung, and prostatic cancer [11,12,13,14]. To a lesser extent, mutations in *TGFBR1* (TβRI, TGFβ Type I receptor) have been revealed in ovary, breast and pancreatic cancer or T cell lymphoma, as well as related to the growth of skin tumors in the Ferguson-Smith disease, a multiple self-healing squamous epithelioma [15,16,17,18]. Besides, *TGFBR1* and *TGFBR2* mutations have also been related to other non-cancerous disorders of clinical relevance, such as the Marfan syndrome or the Loeys-Dietz aortic aneurysm syndrome [19].

The fact that an excessive presence of TGFβ is associated with blood vessel invasion, the promotion of cancer-associated fibroblasts (CAFs), advanced stages of tumors, metastasis, and decreased survival of patients with different types of cancer, points out the reduction of these high levels as one of the main objectives of many targeted cancer therapies [9,20,21]. Notwithstanding, while the establishment of TGFβ as a drug target in cancer seems reasonable, it represents a major challenge, due to a number of factors. On one side, the unintentional inhibition of the tumor-suppressor function of the TGFβ signaling pathway could cause more harm than good [22]. And on the other side, undesirable side effects, such as inflammation, or autoimmunity can develop when TGFβ is unbalanced [23]. No less important are the genetic variations between individuals or the crosstalk of TGFβ signaling cascade with diverse signaling pathways [21,24]. Furthermore, the need for local activation of TGFβ to exert its function makes that not just its abundance in the tumor microenvironment must be controlled, but also its activation process. Among other molecules, the αv-integrins αvβ6 and αvβ8 are specialized in TGFβ activation [25]. Hence, this activation process and the molecules associated represent one more possible target in cancer therapy.

The high incidence, severity, and mortality rates of cancer in the population [26] and the recognized key role of TGFβ in this group of diseases motivated the present research. In this context, the main aim of this work is to elucidate if TGFβ or any of the molecules involved in its synthesis, activation, or signaling pathway could represent an effective and safe therapeutic target in cancer treatment. The systematic review performed revealed that many different therapeutic strategies are being investigated in this sense—many of them offering promising prospects. The most noteworthy are: Antisense oligonucleotide-based therapeutics for gene expression regulation, small molecule inhibitors, receptor traps to prevent ligand-receptor interactions, or molecules that impede TGFβ activation, or signaling. The combination of therapies targeting different processes of carcinogenesis showed improved results over the use of a single strategy.

To enable a better understanding of the mechanism of action of the aforementioned strategies, a brief summary on the synthesis, secretion, activation, and signaling of TGFβ is presented below.

## 2. Synthesis and Secretion of TGFβ

TGFβ is a pleiotropic multifunctional cytokine that belongs to a superfamily of ubiquitous cell growth factors that includes activins, inhibins, Nodal protein, bone morphogenic proteins (BMPs) or the anti-Müllerian hormone [21,27]. The TGFβ family itself refers to a series of growth factors structurally related and codified by 33 genes. Among the different isoforms of TGFβ, the most important in mammals are TGFβ1, TGFβ2, and TGFβ3. These three isoforms present 75% of homology and similar signaling through the same receptor complex, although they exert non-redundant functions and are differentially expressed in specific cells and tissues [28].

**Figure 1:**
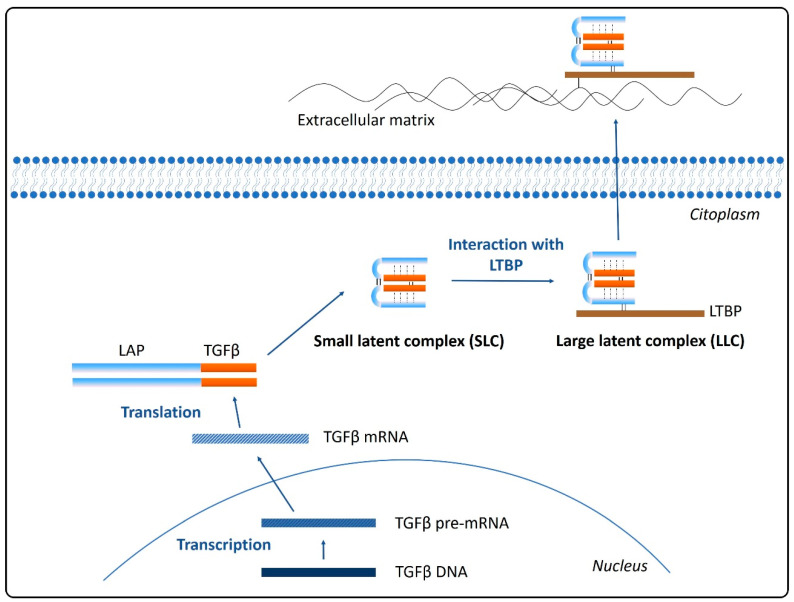
Schematic representation of transforming growth factor β (TGFβ) synthesis and secretion.

Translation from TGFβ mRNA provides a dimeric pro-peptide (pro- TGFβ) that contains the growth factor and the latency-associated peptide (LAP), linked to each other through a non-covalent bond to form the small latent complex (SLC). This precursor is further processed previously to its secretion from the cell by the interaction of the LAP with a latent TGFβ-binding protein (LTBP), producing a large latent complex (LLC). LTBPs are glycoproteins that act as chaperons for pro-TGFβ, mediating its folding and secretion. Therefore, the ubiquitously secreted TGFβ will be anchored to the extracellular matrix and stored in an inactive state by means of interactions between LTBP and fibronectin and fibrillin [28,29] (Figure 1).

## 3. TGFβ Activation and Signaling

**Figure 2:**
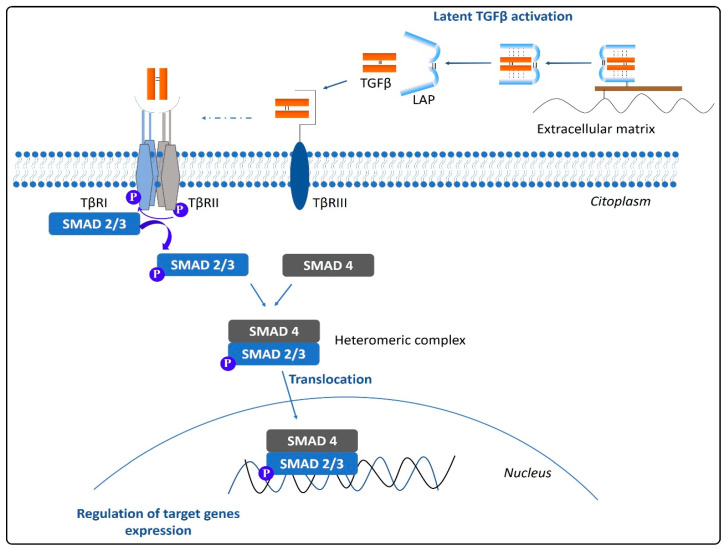
Schematic representation of latent TGFβ activation and the canonical TGFβ signaling pathway.

Prior to interact with its receptors and exert its biological effects, TGFβ requires to be activated. The activation process includes the release of the LLC from the extracellular matrix and the proteolysis of the LAP by the action of different molecules. Latent TGFβ activation is produced by metalloproteinases MMP-2 and MMP-9, thrombospondin, or integrin αvβ6, among others. Moreover, the heat or a decrease in the pH can produce the activation of latent TGFβ [21]. Once activated, TGFβ is available to interact with specific cell surface receptors that will trigger a signaling cascade capable of modifying the cell function (Figure 2). TGFβ family ligands signal by assembling a transmembrane serin/threonine kinase hetero-tetrameric receptor complex consisting of two type I receptor (TβRI or ALK-5) units and two type II receptor (TβRII) units [30]. Besides, auxiliary coreceptors also exist, the so-called type III receptors (TβRIII), which control the access of TGFβ to the signaling receptors TβRI and TβRII. The TβRIII, also known as betaglycan, mediates the interaction between TGFβ and TβRII by inducing a conformational change in the receptor [31]. Through binding the extracellular domain of TβRII in the first instance and that of TβRI afterward, TGFβ provokes the internal kinase domains rapprochement and the acquisition of the specific conformation required to trigger the intracellular signaling cascade. TβRI is thereby phosphorylated, and the signal propagates via two independent pathways: The canonical SMAD-dependent pathway and the non-canonical pathway independent of SMADs [32]. In the canonical pathway, the activation of TβRI results in the phosphorylation of specific receptor-regulated SMADs (R-SMADs), SMAD2 and SMAD3, that will form a heteromeric complex with the common mediator (co-SMAD) SMAD4 and will translocate to the nucleus [33]. There, the SMAD complex associates specifically with the SMAD-binding element (SBE) found in the genome to regulate the transcriptional response. The R-SMADS not only regulate the transcription, but can also modulate the post-transcriptional biogenesis of micro-RNAs. By doing so, R-SMADs enable a quick change in gene expression in response to extracellular stimuli [34]. Conversely, the inhibitory SMAD7 (I-SMAD) can disable the TGFβ signaling pathway by degrading the TβRI receptor, inhibiting the phosphorylation of R-SMADS by TβRI, or by blocking the complex R-SMAD/co-SMAD [30,35]. It is worth noting that other members of the TGFβ superfamily, i.e., BMPs, even if binding to specific receptors, also signal via SMADs. While TGFβ signals via SMAD2/3 and BMPs via SMAD1/5/8 phosphorylation, all these activated SMADs require the subsequent interaction with the common mediator SMAD4 to form the complex that will translocate to the nucleus to modulate gene transcription. Therefore, TGFβ and BMP signaling restrict each other by competing for limited SMAD4 [36]. This opposing role between these two pathways has been suggested to have an implication in cancer progression [37].

In addition, in the non-canonical pathway, the activated TGFβ receptor complex can signal through other factors, such as TNF, TRAF4, TRAF6, MAP3K7, p38MAPK, GTPases Rho family, ERK, JNK, or NF-ƙB [21]. The combination of both canonical and non-canonical pathways will determine the cell response to TGFβ signaling. Furthermore, many other intracellular signaling pathways can interfere with the TGFβ signaling cascade: PI3K-AKT, Wnt, Hedgehog, Notch, IFN, or RAS, among others [38,39,40]. Considering the complexity of the TGFβ intracellular signaling and the vast number of molecules that can affect it, we can assume that any change in the homeostasis of this factor could potentially contribute to crucial events in the cell response with varying degrees of biological relevance.

## 4. Results

### 4.1. Search Results

**Figure 3:**
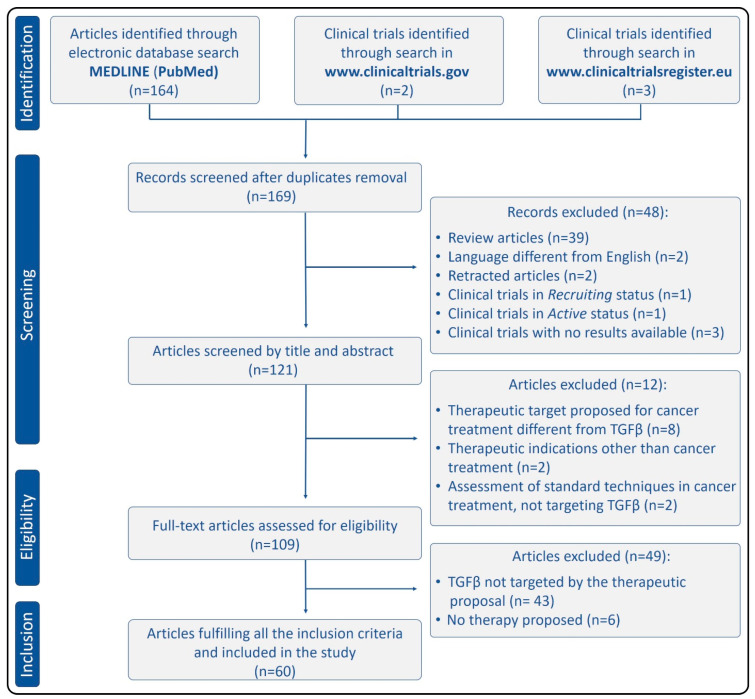
Systematic review workflow.

The initial bibliographic search through PubMed, fulfilling the inclusion criteria related to species and date of publication, produced 164 articles. After removing review articles (*n* = 39) and those written in a language different from English (*n* = 2), 123 results were obtained. Two of them had been retracted, thus they were not included in this study. The relevance of the remaining 121 results was evaluated by analyzing the title and abstract. The potentially relevant articles (*n* = 109) were full-text evaluated, producing 60 articles that were included in this systematic review. Two clinical trials relating to TGFβ and cancer were found through the search engine on the American website www.clinicaltrials.gov. One of them (NCT03834662) was in “recruiting” status, while the other one (NCT02423343) was in “active” status at the moment of the search. Any of the clinical trials are displayed by the official European website www.clinicaltrialsregister.eu (EudraCT numbers: 2015-002093-20, 2011-005983-12, and 2010-022338-10) had results available at the time this study was undertaken, thus they were not included in this review. Figure 3 summarizes the search results using the Preferred Reporting Items for Systematic Reviews and Meta-Analyses (PRISMA) flow diagram [41].

### 4.2. Review of Eligible Studies

Most of the articles analyzed focused on the inhibition of the overexpressed pathways or the activation of the protective ways related to TGFβ that are commonly suppressed in cancer. The issue has been addressed by a wide variety of strategies that have been categorized and commented upon.

#### 4.2.1. MicroRNAs

The use of microRNAs (miRNAs) is the most widespread therapeutic strategy proposed among the articles included in this systematic review. Despite being considered functionless for a long time, the role of these RNAs in diverse pathologies has been extensively proven. miRNAs are aberrantly expressed in cancer cells and tissue, contributing to their development and progression. miRNAs are small (13–25 nucleotides), non-coding RNAs that regulate the gene expression at the post-transcriptional level by linking to the 3′-UTR region of the target mRNA [42,43]. The double-stranded heteroduplex formed blocks the translation to protein and mediates a decrease in the target gene expression [44]. In this way, miRNAs silence or degrade the mRNA molecule. The main limitations in applying miRNAs as a therapeutic strategy are the instability of antisense molecules and the difficulty to specifically reach and enter the cancer cells. Furthermore, the fact that a miRNA can target mRNAs from different genes and a mRNA can be regulated by several miRNAs can pose a problem. Nevertheless, many antisense oligonucleotides targeting TGFβ are in preclinical or clinical development stages [38]. With regard to the articles included in this systematic review, 17 of them employed miRNAs as a therapeutic approach in cancer treatment. More precisely, the following miRNAs were investigated: miR-143-3p [43,45], miR-124 [46], miR-155 [47,48,49], miR-520c [50], miR-17-5p [51], miR-323-3p [20], miR-367 [52], miR-106b [53], miR-455-3p [54], miR-592 [55], miR-153 [56], miR-16 [57], miR-3591-5p [58], and miR-27a [59]. Table 1 comprises the most important data in relation to these studies.

In the research by Guan et al. [43], the TGFβ/miR-143-3p/cystatin axis is proposed as a therapeutic target in cancer treatment. For the first time, the tumor suppressor miR-143-3p was shown to diminish the expression of cystatin, an oncogene that is significantly overexpressed in ovarian, bladder, hepatocellular, and colorectal cancer. In this case, TGFβ contributes to induce mature miR-143-3p that will silence the expression of cystatin. The article published by Cheng et al. [45] on the role of miR-143 sheds some light on the mechanism by which TGFβ acts as a tumor suppressor in healthy tissue and as a tumor-promoter in early carcinogenesis through the loop miR-143/SMAD3/ TGFβ. While TGFβ can increase the reduced expression of miR-143 in non-small cell lung cancer (NSCLC), the overexpression of this miRNA inhibits the endogen expression of SMAD3 at the post-transcriptional and post-translational level. A decreased expression of SMAD3 will lead to the reduction of SMAD2/SMAD3 complex that will consequently restrict the TGFβ signaling pathway. The antitumor effect of the overexpression of miR-143 is shown not only in vitro, but also in vivo in this research article, resulting in a reduced size and volume of xenografts from miR-143-overexpressed NSCLC cells in nude mice [45]. Another miRNA whose expression is decreased in NSCLC is miR-124. miR-124 can reverse EMT by suppressing SMAD4 [46].

In three of the selected articles, miR-155 has been proposed as a therapeutic target (Table 1). miR-155 is associated with the growth of cancer cells and metastasis through binding the 3′-UTR region of TβRII mRNA and the subsequent inactivation of this receptor. Qu et al. proposed miR-155 repression and TβRII overexpression as a strategy in gastric cancer [47]. Nevertheless, several issues arise in this approach. First, the role of TβRII in tumorigenesis is controversial, since it can act either as tumor inhibitor [47] or as proto-oncogen [60]. Secondly, TβRII is not the only target of miR-155. Many other molecules with a relevant role in gastric cancer progression are regulated by miR-155, such as c-myc, SMAD1, the signal transducer and activator of transcription 1, calcium-binding protein 39, CXC chemokine receptor 4, or the carbonic anhydrase 9 [61,62,63]. Lastly, the role of miR-155 in the tumor microenvironment is highly dependent on the cancer type. While the overexpression of miR-155 is found in gastric [47] or colon cancer [48], its suppression has been linked to cancer aggravation in melanoma, Lewis lung carcinoma, EL4 lymphoma, or prostate cancer models [49,64,65]. Moreover, the hyperactivation of the TGFβ/SMAD pathway in miR-155-/- mice promotes carcinogenesis [48]. In addition, miR-155 not only inactivates TβRII, but also inhibits the expression of endogen and TGFβ-induced SMAD2/3 [49].

Other miRNAs investigated in the articles comprised in this review that target the receptor TβRII are miR-520c [50] and miR-17-5p [51]. miR-520c expression is decreased in glioma compared to healthy tissue and linked to a lower survival rate in the patients. The suppressor role of miR-520c is demonstrated in vitro, but no in vivo experiments are presented to confirm its effectiveness in cancer treatment in this article [50]. Unlike miR-520c, the miR-17-5p expression is increased in cancer tissue. In this research, the downregulation of TβRII expression by miR-17-5p is investigated in gastric cancer. The loss of TβRII allows cells to escape the inhibitory effect of TGFβ, and promotes cell growth and migration [51]. For its part, miR-323-3p targets and blocks SMAD2 and SMAD3 expression. The reexpression of miR-323-3p is proposed as a therapy against metastasis in pancreatic ductal adenocarcinoma (PDAC) patients [20]. An opposing effect in this type of cancer is exerted by miR-367. Unlike miR-323-3p, miR-367 is increased in PDAC [52]. miR-367 acts by reducing SMAD7 expression, the I-SMAD that inhibits the TGFβ signaling cascade [52]. Moreover, miR-106b functions in a similar way by targeting SMAD7 in gastric cancer [53]. However, the proposed therapies targeting miR-367 or miR-106b are not delivered in these articles [52,53], hence their efficiency and safety cannot be evaluated.

An oncomiR for which a treatment has been tested is miR-455-3p. Liu et al. administered an antagomiR to inactivate the TGFβ/SMAD and the Wnt/β-catenin signaling pathways in esophageal squamous cell carcinoma (ESCC) [54]. The antagomiR-455-3p produced the sensitization of cisplatin-resistant cancer cells and inhibited tumor growth.

A miRNA that requires a careful analysis prior to being considered as a therapeutic target is miR-592. The expression of miR-592 is significantly decreased in breast cancer and inversely correlates to the proto-oncogene TGFβ2 expression [55]. However, despite the tumor suppressor capacity of miR-592 in breast cancer [55], small cell lung cancer [66], or hepatocellular carcinoma (HCC) [67], this miRNA plays an oncogenic role in prostate [68], and colorectal cancer (CRC) [69]. Moreover, miR-153 reduces the expression of TGFβ2 and acts as a tumor suppressor in osteosarcoma [56], albeit the administration of a therapy targeting this miRNA has not been investigated thus far.

Furthermore, miRNAs have been linked to the expression of EMT-associated genes, as for miR-16 [57], or to radiotherapy-induced EMT in cancer treatment. In this sense, miR-3591-5p is the highest expressed miRNA in response to radiation and has been proposed as a therapeutic target in cancer treatment [58]. miR-3591-5p inhibition has been proven effective in preventing the effects of the radiation regarding morphological changes in cells, the expression of the mesenchymal markers vimentin and α-smooth muscle actin, and the activation of TGFβ signaling in lung cancer [58]. Another miRNA that is overexpressed in lung cancer and has been proposed as a therapeutic target among the articles included in this review is miR-27a [59]. miR-27a acts as an oncogen by repressing the expression of the tumor suppressors SMAD2 and SMAD4. While this article contributes to enhancing the scientific knowledge on the molecular mechanisms of miR-27a on TGFβ signaling regulation, no treatment is administered to test its potential effectiveness.

#### 4.2.2. Small Interfering RNA

Other studies included in this systematic review have also made use of gene silencing by non-coding RNA as a therapeutic tool. In this case, with small interfering RNA (siRNA). siRNAs consist of 19–21 nucleotides and operate at different levels that include chromatin remodeling, translation inhibition, or mRNA degradation [70]. While miRNA and siRNA are both capable of silencing a gene, two main differences exist between them. As alluded to earlier, miRNAs can regulate the expression of multiple mRNAs. On the contrary, siRNA prevents the expression of a specific target mRNA, due to perfect complementarity with its sequence [70]. Additionally, miRNAs are endogenous single-stranded RNAs, while siRNAs are considered double-stranded RNAs from the exogenous origin, being intracellular delivery one of the biggest challenges in their use [71].

Li et al. successfully silenced SMAD4 in doxorubicin-resistant colon cancer cells by using a lentivirus vector [72]. Doxorubicin treatment provokes the increase of TGFβ levels, EMT, and resistance after long-term administration. However, no in vivo results are shown in this article that could lead to future clinical trials.

RNA interference has been employed in two more articles, but in the form of short hairpin RNA (shRNA). The presence of these hairpins confers RNA major stability and low degradation rate. Kang et al. assayed the combined therapy with sorafenib and a shRNA against TGFβ1 and TGFβ2 in HCC [73]. The administration of the shRNA contributed to the sensitization of sorafenib-resistant cancer cells by recovering the phosphorylation of p38, which induces cell death. The in vivo experiments in a xenograft model in nude mice did not revert the drug resistance completely, however it significantly improved the survival rate of the animals compared to sorafenib alone. Meanwhile, Wang et al. demonstrated that the combined administration of a TβRI inhibitor (SB431542) and a shRNA against SMAD2/3 procured a reduction in the ability of sphere-formation by CD51+ CRC cells, cell motility, and tumor formation [74].

#### 4.2.3. Long Non-Coding RNA Activated by TGFβ (lnc-ATB)

Besides miRNAs and siRNAs, other non-coding RNAs with a significant role in transcriptional and post-transcriptional gene expression regulation in many diseases, including cancer, are long non-coding RNAs (lncRNAs). LncRNAs are transcripts that exceed 200 nucleotides in length [75,76]. Generally, these RNAs are not translated into proteins, but some of them can produce small functional peptides [77]. An interesting characteristic of lncRNAs is their highly restricted expression pattern in time and space. They are expressed with a tissue/cell type/stage of development specificity much higher than coding RNAs [75]. Dysfunctional lncRNAs have been related to tumor growth and can act as oncogenes [78]. In this process, the interaction lncRNA-miRNA-mRNA is essential. LncRNAs can compete with a miRNA for the same mRNA, sequester a miRNA, or produce different miRNAs [79].

Four of the reviewed studies suggested lncRNA activated by TGFβ (lnc-ATB) as a therapeutic target. Zhang et al. demonstrated that miR-141-3p is underexpressed due to the overexpression of lnc-ATB in breast cancer tissue. The silencing of lnc-ATB caused the inhibition of cell migration and invasion, EMT, and metastasis [80]. Similar effects were achieved after lnc-ATB silencing in cervical cancer [81], and renal cell carcinoma [82]. Zhai and Xu found that the suppression of miR-126 mediated by the overexpressed lnc-ATB increases KRAS mRNA and protein, consequently activating PI3K/AKT and mTOR pathways in bladder cancer [83]. Despite the positive outcomes achieved by lnc-ATB silencing, this therapy has not been tested in vivo in any of these articles.

#### 4.2.4. Small Molecule Inhibitors (SMI)

There is a substantial number of small molecules that have been shown to be valuable for cancer treatment in the reviewed literature. These molecules, usually ATP-mimetic, specifically target the type I TGFβ receptor TβRI and block the SMAD2 and SMAD3 phosphorylation. This is the case of galunisertib (LY2157299), a competitive inhibitor of TβRI developed by Eli Lilly and Company (Indianapolis, IN, USA). This molecule significantly suppressed the constitutive activation of the TGFβ/SMAD signaling in the highly malignant keratin 19-positive HCC cancer stem cells in the reviewed study published by Kawai et al. [84]. Notwithstanding, it should be noted the importance of personalizing cancer therapy. Agarwal et al. highlighted this issue after finding contrasting reactions to galunisertib treatment in HCC patients [85].

Another TβRI inhibitor analyzed in one of the reviewed articles is SB431542, a drug candidate developed by GlaxoSmithKline. This compound was able to inhibit the mobility and invasiveness of HCC cells with mitochondrial DNA depletion, as well as the expression of mesenchymal markers, TGFβ, the gene Snail, or the phosphorylation of SMAD2/SMAD3 [86]. SB431542 has also been tested as a treatment for NSCLC [87] or ovarian cancer [43], showing its capability to avoid the effects of TGFβ1 and revert EMT. However, its effectiveness in clinical applications has not been proved in these articles.

Another SMI with a high affinity for TβRI that was investigated in HCC in one of the articles reviewed is EW-7197. The administration of this compound successfully reduced the volume of tumors and attenuated the intrahepatic metastasis in an in vivo xenograft model in mice [88]. LY2109761, a selective inhibitor of SMAD2, was able to decrease mesenchymal cell proliferation and survival. Cell death was further increased when concomitantly administered with radiotherapy. This study confirmed the beneficial effects of this combined therapy previously shown in breast cancer [89] and glioblastoma [90].

A small peptide, p234, was assayed in triple-negative breast cancer. P234 is an antagonist of KiSS1, a downstream target of the canonical TGFβ/SMAD2 pathway in this cancer type. The blocking of KiSS1 impaired in vitro the TGFβ-mediated cell invasion [91]. Moreover, the effect of IM-412 was also investigated in triple-negative breast cancer cells [92]. Apart from affecting the SMAD-dependent pathway by preventing SMAD2/3 phosphorylation, IM-412 also involves the non-canonical pathways by suppressing p38MAPK, AKT, and JNK phosphorylation. In this study, the capacity of IM-412 to block the tyrosine kinase receptor FGFR3 activation is also observed. Since this receptor has been proven to contribute to metastasis, the effect of IM-412 on cell migration and invasiveness could be mainly due to alternative pathways besides TGFβ.

A direct target of TGFβ signaling that has been inhibited in cancer treatment is PTP4A3 [93]. The loss of signaling by TGFβ during cancer progression causes the activation of the cell survival pathway PI3K/AKT mediated by PTP4A3, which can selectively promote metastasis. PTP4A3 is overexpressed and related to poor survival in patients with different types of cancer: breast, ovarian, liver, colon, or gastric cancer [94,95,96]. The PTP4A3 inhibitor here employed was pentamidine, an FDA-approved antifungal, and antiprotozoal agent. In the reviewed article, the treatment of carboplatin- and paclitaxel-resistant endometrial cells with pentamidine repressed cell viability in a dose-dependent manner [93]. This is consistent with the anticancer activity of pentamidine previously shown in human cancer lines and preclinical models of human cancers [97].

The human transcription factor AP4 (Activating protein-4) is another of the molecules proposed in one of the articles included in this review to be administered in NSCLC [98]. AP-4 can avoid SMAD2 phosphorylation, and hence, decrease p21 levels to prevent the effects of TGFβ on cell growth. TGFβ1 can, in turn, activate AP-4 transcription. This study is, once more, performed in cell cultures. In spite of the relevance of the results drawn from these studies, an in vitro to in vivo extrapolation is not possible without the appropriate experiments.

Likewise, the use of SMIs has been proved helpful in overcoming drug resistance. Thus, even if not considered a therapy itself, the combined administration of these molecules with anticancer drugs enhances the effectiveness of the latter. Several studies have revealed the fundamental role of TGFβ in drug resistance, and a correlation between EMT and resistance to anti-HER2 drugs in HER2-positive breast cancer cells has been described. In this regard, the SMAD3 inhibitor SIS3 (Sugar insensitive 3) has been tested as a suppressor of anti-HER2 drug resistance. In addition to this capacity, SIS3 also restored sensitivity to trastuzumab in resistant cells [99]. Regarding erlotinib-resistance, the dual TβRI and TβRII selective inhibitor LY2109761 developed by Eli Lilly and Company was also assayed in lung cancer cells in one of the reviewed articles [100]. This molecule blocked the TGFβ signaling by preventing SMAD2 phosphorylation, along with a reduction in the overexpressed levels of the protein kinase Cα (PKCα) mRNA in erlotinib-resistant cells. The TGFβ/PKCα route is associated with the expression of genes related to the mesenchymal phenotype.

Beyond the therapeutic proposals based on the TGFβ canonical pathways, two of the studies included in this review proposed TAK1 as a drug target in pancreatic and breast cancer treatment [101]. A derivate from imidazopyrazine presented a significant in vivo antitumor effect in mice subjected to xenotransplantation with human mutant KRAS-dependent CRC cells [101]. Additionally, the TAK1 inactivation by the inhibitor 5Z7 (5Z-7-oxozeaenol) improved the efficiency of the treatment with Taxol (paclitaxel) in ovarian cancer cells. Furthermore, this molecule inhibited tumor growth in vivo in a xenograft model in mice [102]. The synergistic effects of 5Z7 and Taxol were demonstrated, since the treatment with only one of them presented no improvements compared to the non-treated group.

#### 4.2.5. Receptor Traps

Receptor traps are recombinant peptides that mimic the extracellular domain of TGFβ receptors. These molecules are, unlike the corresponding signaling receptors, not anchored to the cell membrane. Thus, they are soluble and able to sequester the ligand TGFβ, consequently preventing their intracellular signaling and biological effects.

In the reviewed literature, just one of the articles assayed this strategy. Qin et al. developed a novel TGFβ receptor trap containing the domains of TβRII and TβRIII [103]. In this article, the receptor trap completely blocked the TGFβ–TβRII binding and hence TGFβ1 and TGFβ3 signaling in cultured epithelial cells. More importantly, the systemic administration of this TGFβ receptor trap inhibited tumor cell proliferation and the invasion potential of tumor cells in high grade prostatic intraepithelial neoplasia lesions in a prostate-specific knockout mice model of PTEN, one of the most common tumor suppressor genes altered in prostate cancer [104].

#### 4.2.6. Strategies Targeting Latent TGFβ Activation

The main role of αvβ6 integrin in TGFβ activation from the large latent complex in which it is stored in the extracellular matrix, indicates that this integrin could be an interesting therapeutic target in many diseases, including cancer. αvβ6 integrin activates TGFβ by degrading the LAP, and then the active cytokine will activate CAFs first and cancer cells afterward. The knockdown of αvβ6 integrin disrupted this process in CRC cells. In this study, also the compound AMD3100, an antagonist of the CXCR4 (chemokine receptor type 4) axis, was able to inhibit CAFs-induced CRC cells invasion and to completely prevent the effects related to tumor progression [105]. Such is the importance of αvβ6 integrin in TGFβ activation that GlaxoSmithKline initiated a program to search for small molecule αvβ6 selective RGD-mimetics. As a result, compound 1 was identified and demonstrated to bind with extreme affinity and selectivity for the αvβ6 integrin. Nevertheless, the biological effects of this drug candidate in cancer prevention were not assayed in this article [106].

#### 4.2.7. Natural Compounds

Many substances of natural origin have been tested in cancer treatment. Several references included in this review show the administration of compounds from different natural sources that target TGFβ or any of the molecules involved in its signaling. They include the chemical compounds resibufogenin [107], resveratrol [108], nobiletin [109], glabridin [110], or the medicinal plants *Scutellaria baicalensis* and *Fritillaria cirrhosa* [111].

Resibufogenin is one of the major active compounds of the HuaChanSu, the dried venom secreted from the skin of the giant toad (*Bufo bufo gargarizans* Cantor and *Bufo melanostictus* Schneider) that has long been used in the traditional Chinese medicine in cancer treatment [112,113,114]. Liu et al. provided evidence that resibufogenin displays selective cytotoxicity against pancreatic cancer cells in a dose- and time-dependent manner. The experiments in vivo also provided positive results in a xenograft model using athymic nude mice, with a dramatically decrease of the tumor mass and volume, and no signs of systemic toxicity [107].

The effect of resveratrol, a stilbenoid that is traditionally known for its antioxidant and anti-inflammatory properties, has been analyzed in breast cancer metastasis in the article published by Tang et al. [108]. Resveratrol acts by activating SIRT7 deacetylase activity, which is significantly downregulated in breast cancer lung metastases. SIRT7 deacetylates and promotes SMAD4 degradation; hence, antagonizes TGFβ signaling and inhibits metastasis. The treatment with resveratrol in Balb/c mice injected with 4T1 cells caused an important reduction in the metastatic nodules and reached an overall survival at 30 days of 100% compared to 50% in the vehicle group.

Nobiletin, a flavonoid with multifunctional effects found in Citrus fruits, was tested in NSCLC [109]. This compound successfully prevented TGFβ1-induced EMT as well as the migration, invasion, and cell adhesion in vitro. In vivo, nobiletin not only inhibited the growth of the tumor and the metastatic nodules in the lungs of nude mice, but also reversed EMT in mice bearing A549-Luc xenografts.

The administration of glabridin has also been suggested as a potential treatment strategy in breast cancer therapy. Glabridin is an isoflavane present in the root extract of licorice (*Glycyrrhiza glabra*) that was effective in reducing the cancer stem cell-like properties through the miRNA-148a/ TGFβ-SMAD2 pathway in human breast cancer cells both in vitro and in vivo. Consequently, tumor growth and mesenchymal characteristics were attenuated in mouse xenograft models [110].

Regarding the use of medicinal herbs, the treatment with *Scutellaria baicalensis* (SB) and *Fritillaria cirrhosa* (FC) extracts was assayed in endometrial cancer in one of the reviewed articles. Both extracts were able to diminish the expression of the three isoforms of TGFβ, TβRI, TβRII, and SMADs in human endometrial cancer cells. The results obtained by Bokhari and Syed suggested that SB and FC extracts prevent endometrial cancer cell proliferation, invasion, and metastasis by inhibiting TGFβ/SMAD3-mediated EMT and by downregulating TGFβ-activated integrins and FAK expression [111]. However, all the experiments in this article were performed in vitro, and no preclinical studies were shown.

#### 4.2.8. Drugs Approved for Other Diseases

Likewise, the effect of previously marketed drugs for other therapeutic indications has been investigated in the search for a successful cancer treatment. Besides the SMI pentamidine previously commented, the ionophoric coccidiostat and antibiotic salinomycin was also evaluated in cancer therapy in one of the articles included in this review [115]. PDACs are characterized by a dense stroma that acts as a physical barrier to chemotherapy. Here, the authors proposed a drug delivery system based on poly (lactic-co-glycolic acid) (PLGA) nanoparticles to ease the passing of salinomycin through the pores of tumor vessels and to prolong the drug release around the tumor cells in an orthotopic model of pancreatic cancer. This therapy induced apoptosis mainly in the tumor nest, achieving a 52% reduction in tumor size in comparison to the control group. The authors conclude that salinomycin attenuates tumor development by boosting TGFβ/TβRII signaling pathway in tumor microenvironment and consequently reverting EMT [115].

Sitagliptin is another drug that has been tested in papillary thyroid carcinoma (PTC) in the reviewed literature. Sitagliptin is a dipeptidyl peptidase-4 (DPP-4) inhibitor used to treat type 2 diabetes and commercialized by Merck and Co. under the name Januvia. Since there exists evidence of overexpression of DPP-4 in malignant thyroid neoplasms as well as alterations in its expression and activity in several solid and hematological malignancies, Lee et al. aimed to analyze the therapeutic potential of DPP4 inhibition in PTC [116]. The authors demonstrated that the TGFβ signaling pathway is involved in the regulatory effects of DPP4. The pharmacological and genetic inhibition of DPP4 suppressed colony formation, cell migration, and invasion of thyroid cancer cells in vitro. More importantly, the treatment with sitagliptin significantly reduced the tumor growth in a mouse xenograft model, with no influence in the body weight or blood glucose levels. In addition, sitagliptin produced a decrease in *TGFBR1* expression among xenograft tumors.

The role of simvastatin, a statin used to lower blood cholesterol levels, has also been evaluated as an anticancer drug. Given that statins exhibit cancer chemopreventive properties, Shang et al. tried to elucidate the effects of simvastatin in lung cancer [117]. This drug inhibited cell proliferation and downregulated the expression of TβRII. They also found that simvastatin suppressed the activation of ERK (extracellular signal-regulated kinase), a protein kinase that participates in a SMAD-independent signaling pathway. Therefore, simvastatin could exert antitumor effects through two different pathways.

#### 4.2.9. Strategies Targeting Molecules with an Effect on TGFβ Regulation

Among a vast quantity of therapeutic strategies proposed in the reviewed articles, the modulation of the expression of different molecules capable of interfering with the TGFβ signal transduction has also been considered.

Although the crosstalk between TGFβ and STAT pathways had previously been proposed, the direct interaction of the transcriptional factor STAT1 with TGFβ receptors was shown for the first time in the article by Tian et al. included in this review [118]. The overexpression of STAT1 prevented the suppressor role of TGFβ on cell proliferation, migration, and invasion in epithelial ovarian cancer cells. Thus, the suppression of STAT1 was suggested as a potential therapeutic strategy in ovarian cancer treatment, albeit no in vivo experiments were performed in this article.

Another transcription factor proposed as a target in glioma treatment was HOXC10 (Homeobox protein Hox-C10). HOXC10 expression is increased in tumor tissue and promotes cell proliferation, migration, and invasion, as well as colony formation, an aggressive cell phenotype, and inducement of immunosuppressor gene expression. In this article, Li et al. demonstrated that shRNA silencing of HOXC10 reduced the expression of TGFβ2, among other factors. HOXC10 is proposed as a non-redundant therapeutic target to be modulated in combination with those controlling the immune checkpoints [119]. In the same way, the silencing of HMGA2 (high mobility group protein A2) has been suggested as an option in cancer treatment. HMGA2 is a transcription factor that is overexpressed in tumor development. Conversely, its silencing inhibited the TGFβ/SMAD pathway and thus, suppressed cell proliferation, migration, and invasion, while promoting apoptosis in prostate cancer cells [120].

TRIM59 (Tripartite motif protein 59) is another molecule investigated in one of the reviewed articles and overexpressed in breast cancer cells. The silencing of TRIM59 by means of siRNA reduced p-SMAD2 and inhibited cell proliferation, migration, and invasion in vitro. Furthermore, TRIM59 silencing diminished tumor size in a tumorigenic model in nude mice [121].

Additionally, the silencing of the transmembrane protein TMEM45 reduced the tumor cell proliferation, adhesion, and invasiveness in ovarian cancer. The mechanism involves the TGFβ pathway, since TMEM45 silencing provoked a reduction in TGFβ1 and TGFβ2 mRNA and protein. Nevertheless, RhoA and ROCK2 levels were also affected by TMEM45 silencing, both associated with alterations in the cell invasion and adhesion capacities [122].

Likewise, the silencing of the overexpressed DRAK1 (Death-associated protein kinase-related apoptosis-inducing protein kinase 1) protein in head and neck cancers was evaluated. Park et al. found that cytoplasmic DRAK1 increases the tumorigenic potential through inhibition of TGFβ1-mediated suppressor activity by directly binding to SMAD3 and interrupting the complex SMAD3/SMAD4 formation. Accordingly, DRAK1 depletion in a xenograft assay led to tumorigenicity suppression [123].

For its part, the deubiquitination enzyme CYLD is capable of inducing different tumor suppressor pathways, and its overexpression inhibits SMAD7-mediated cell invasion in oral squamous cell carcinoma (OSCC). For this reason, CYLD has been proposed as a potential therapeutic target to prevent OSCC metastases [124].

Finally, the competition between TGFβ and BMP7 (bone morphogenetic protein-7) for the common signal transducer SMAD4 has pointed out the latter as a promising drug target in breast cancer treatment. Ying et al. verified that no cross-activation of receptors by TGFβ1 and BMP7 takes place in breast cancer, yet they can affect each other through interaction with the downstream signaling. While TGFβ triggers the signaling cascade through SMAD2/3 and BMP7 through SMAD1/5/8, they both require the binding of these phosphorylated SMADs to SMAD4 to form the SMAD-complex that will translocate into the nucleus. In this article, BMP7 is shown to be able to antagonize the tumorigenic effect of TGFβ1 in breast cancer cells, by reducing the TGFβ1-induced activation of EMT-related genes, cell growth, and metastases [125].

**Figure 4:**
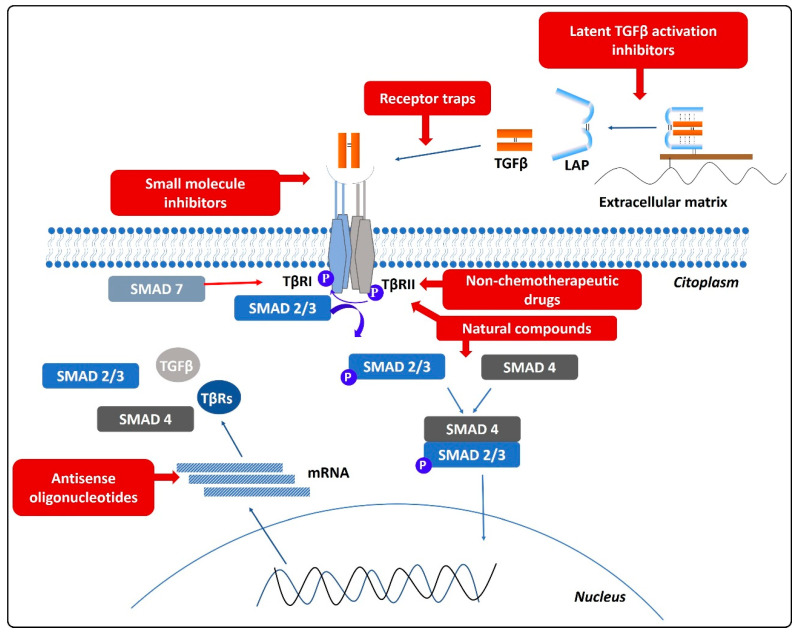
Graphical overview of therapeutic strategies (in red) aimed at TGFβ modulation in cancer treatment. The level at which each strategy is targeted (synthesis, activation, or signaling processes) can be noted.

As a summary, Figure 4 shows the different therapeutic approaches included in the reviewed articles and previously presented.

## 5. Discussion

The systematic review here presented evidence that TGFβ attracts significant interest, and many efforts are being made to modulate this factor in the search for a successful therapy in cancer treatment. The role of TGFβ in many crucial processes and mechanisms related to tumor progression, such as cell proliferation, differentiation, invasiveness, apoptosis, or EMT, has been demonstrated in different cancer types within the reviewed articles. The methodology employed in the vast majority of them consisted of an analysis of the molecular mechanisms related to TGFβ signal transduction and identifying the molecules involved. According to this, a therapy targeting those molecules was proposed. Nevertheless, once the relevance of a specific molecule was proven in cancer development and/or progression, just some studies assayed the suggested therapy in preclinical models. When moving from the lab bench to the clinical setting, the number of publications drastically dropped, and no clinical trials were found in the period considered in this review. The basic research articles included in this review contribute nevertheless to widen the scientific knowledge on the function of TGFβ and related factors in cancer, which brings us closer to an effective and safe therapeutic strategy.

In the race for a cancer cure, most of the members of TGFβ signaling pathway have been proposed as a drug target. In a similar way, the strategies suggested differ greatly among them and operate at different levels, i.e., direct inhibition of TGFβ synthesis or activation, blocking of TGFβ-receptor interaction, or interference with the downstream effectors of TGFβ signaling cascade. Thereby, there exists a very wide range of possibilities to halt cancer progression that have been evaluated.

Gene silencing is the technology most frequently employed in the reviewed literature. This tool has shown favorable outcomes in vitro and in preclinical studies. By contrast, the translation into clinical practice is not presented in any of the articles here included. The main obstacles to siRNA in vivo administration are the fast degradation into the bloodstream and the difficulty to reach the cell interior. This requires the development of appropriate delivery systems. Furthermore, cell-specific targeting is needed, for which viral vectors are usually employed. Beyond representing a risk factor, they provoke an immune response in the patient. This immunological reaction that could be an adverse effect in some cases, could rather contribute to cancer treatment in other ones [126,127]. It is known that TGFβ overexpression can suppress the antitumor immune response system in tumors. However, among several RNA-based anticancer that have entered clinical trials [128,129], some of them terminated earlier, due to immune-related serious adverse events [130]. On the contrary, endorsed by their immunostimulatory activity, some vaccines have been proposed in cancer treatment in the past. The VigilTM Vaccine -a bi-shRNA furin and GMCSF Autologous tumor Cell Vaccine- has reached phase II clinical trials in ovarian cancer (NCT01309230), advanced melanoma (NCT01453361), and colorectal carcinoma with liver metastases (NCT01505166). The furin shRNA blocks furin protein production that, in turn, indirectly decreases the conversion of the proforms of TGFβ1 and TGFβ2 into active proteins. The clinical trial in ovarian cancer was still ongoing at the moment this review was completed, while the other two had been previously terminated, due to a business decision to pursue other indications. Antisense therapy has also been employed to develop the therapeutic vaccine belagenpumatucel-L (Lucanix^®^) for NSCLC treatment [38]. This allogenic tumor cell vaccine consists of the gene modification of four NSCLC cell lines to block their TGFβ secretion. For this purpose, cells were transfected with a human TGFβ2-antisense vector and subsequently injected in patients with stages II to IV NSCLC. The evaluation of belagenpumatucel-L was performed through different clinical trials (NCT01058785, NCT01279798, and NCT00676507) that were completed before the period included in the present review. In the Phase III study, the efficacy of belagenpumatucel-L as a maintenance therapy was analyzed. Notably, an improvement in overall survival within 12 weeks of the completion of frontline chemotherapy—especially in those patients previously subjected to chemoradiation therapy— was observed [131]. Furthermore, trabedersen (also known as AP12009 or OT101) is a synthetic antisense oligodeoxynucleotide specific for TGFβ2 mRNA with the orphan drug designation by FDA that had gone through clinical trials in pancreatic and colorectal neoplasms, melanoma (NCT00844064), anaplastic astrocytoma, and glioblastoma (NCT00761280 and NCT00431561) [21] in a period prior to that considered in this review. The studies in Phase I/II showed that trabedersen is safe, well-tolerated, and increased patient survival [132]. A post-hoc analysis of the Phase II clinical trial (NCT00431561) outcome data in recurrent or refractory high-grade gliomas was performed. It found that, when intratumorally administered as a single agent, trabedersen was able to induce the stabilization of the disease or a durable partial or complete response in the best-case scenario, with a reduction in the tumor volume in more than one-third of the efficacy population [133]. Unfortunately, the SAPPHIRE Phase III trial (NCT00761280) was terminated earlier, due to the inability to recruit the projected patient number and incomplete collection data. In summary, even if RNA-based therapies offer promising therapeutic applications, there seems to be a long way forward, not without difficulties in diverse forms, before a successful and safe RNA-based cancer therapy can be applied.

Another strongly expanding research line in cancer treatment, as it has been shown in this review, is the use of SMIs. One of the compounds that has moved further among the SMIs included in this review is galunisertib. A phase II clinical trial of galunisertib (NCT01246986) that was active when the search was done has recently been completed, and results were posted on ClinicalTrials.gov in August 2020. Part of the results related to this clinical trial had previously been published and showed that galunisertib was well tolerated at the established dosing schedule and its safety profile was relatively benign compared to other treatment options in HCC, including sorafenib [134]. Other clinical studies with galunisertib had been performed in glioblastoma or pancreatic cancer, among others [135]. However, the development of galunisertib by Eli Lilly was discontinued in January 2020 [136].

Similar to the receptor trap assessed in one of the articles included in this review [103], other peptide inhibitors of TGFβ had been previously proposed as a cancer treatment in combination with antitumor immunotherapy [137]. In particular, the TGFβ inhibitor peptide P144 (derived from the extracellular sequence of the human TGFβ type III receptor) successfully impaired tumor growth and increased survival of nude mice implanted with human glioblastoma xenografts [138].

Apart from receptor traps, monoclonal antibodies are also a tool used to block the TGFβ-receptor interaction, though not present in the reviewed literature. The specificity of monoclonal antibodies and their capability to eliminate a ligand excess in the extracellular compartment make them, however, worthy of mention. A series of antibodies against TGFβ (fresolimumab, lerdelimimab, metelimumab, LY2382770), TβRI (PF-03446962), or TβRII (IMC-TR1) have been developed for both neoplastic and non-neoplastic applications, some of them even entering into clinical trials [21,38,139].

Regarding molecules aiming to prevent TGFβ activation, also an antibody anti-integrin αvβ6 had been developed by Biogen Idec prior to the period under consideration in this review. The treatment with this antibody significantly inhibited the xenograft tumor growth in an athymic nude mice model [140].

Meanwhile, the review here presented corroborated that natural chemicals are very promising anticancer candidates. The substances here commented come, hence to join a plethora of compounds from a natural origin that had been previously employed in cancer therapy, e.g., genistein in HCC or berberine in lung cancer [141,142]. Nonetheless, the success of a specific therapy highly depends on the type and stage of cancer. While resveratrol inhibits breast cancer metastases through the activation of the SIRT7 deacetylase activity, the upregulation of this protein has been related to poor prognosis in prostate and CRC [143,144]. Besides, SIRT7 has been shown to be overexpressed in the early stages of breast cancer and diminished gradually later on. Therefore, SIRT7 seems to be dynamically self-regulated in tumor progression, which hinders the understanding of its role in a specific case [145].

In respect of the use of drugs with different therapeutic indications, the angiotensin II receptor antagonists losartan and candesartan or the anti-fibrotic pirfenidone, have also proved effective in TGFβ signaling reduction, albeit the molecular mechanisms of this action and their role in cancer treatment are still ignored [146,147].

Besides the type and stage of cancer, the individualized response of patients to TGFβ blocking is of paramount importance. Many of the diseases treated with TGFβ inhibitors, cancer included, are highly complex and have a strong genetic influence. Thus, a previous selection of potential candidates for TGFβ-targeted pharmacological treatments should be considered. Ideally, those individuals in which TGFβ drives the progression of the disorder should be addressed, while this kind of treatment should be disregarded in the subset of patients particularly vulnerable to inflammation or specific vascular conditions.

Another important aspect is the use of combination therapies. Better outcomes are achieved when therapies against TGFβ are used together with therapeutic strategies targeting other mechanisms related to carcinogenesis. Hence, the administration of immune checkpoint inhibitors could contribute to counteracting the TGFβ-mediated host immune surveillance suppression. A higher number of active T cells will provide a greater response that will kill cancer cells more efficiently and will reduce the growth of metastatic tumors [148,149]. This therapeutic approach, together with the use of suppressors of mesenchymal properties or TGFβ signaling inhibitors, potentially offers more meaningful outcomes than a single strategy. Despite this, the mechanism through which therapies work and their effects on the organism always require careful consideration. This is especially important when radiation or chemotherapy are applied, since they can produce an increase in circulating TGFβ1 levels that could promote metastasis [150]. Combined therapy becomes particularly relevant when drug resistance is acquired. Several articles included in this review reported the administration of anticancer agents together with anti- TGFβ shRNA [73] or SMIs [99,100]. Acquired resistance is highly influenced by the tumor stroma, namely CAFs. CAFs originate from normal fibroblasts, which have suffered a transition to SMA-expressing myofibroblasts. This transition occurs under stimulation by TGFβ1 secreted by cancer cells in response to anticancer drug treatment [151,152]. CAFs are perpetually activated and boost the extracellular matrix production and cell contraction. Consequently, the interstitial fluid pressure rises, and drug delivery is impeded in the tumor tissues. Besides, TGFβ-driven EMT favors tumor cell dissemination and further hampers antitumor therapies [153]. In this sense, TGFβ blockade would disrupt the bi-directional crosstalk between stromal and cancer cells, which allows the tumors to amplify a drug-resistant niche [151].

One of the main limitations of the present study is that the search period considered did not include some of the pharmacological approaches that have moved further in clinical trials. The required duration of a clinical trial since a potential treatment is proposed until the study is completed can extend to several years—this will lessen the chance to be found through a search, such as the one performed here. Even so, the mention of those studies in the articles actually included allowed the discussion on the outcomes they provided. Notably, any of these studies include a TGFβ-targeted therapeutic strategy that currently represents the first-line treatment for cancer patients.

Future research directions point towards applying more recent techniques with clinical applicability. CRISPR/Cas9 gene-editing technology has revolutionized the genome, transcriptome, and epigenome modifications and has begun to yield its first clinical results, namely in cancer [154]. This tool could overcome some of the current limitations of gene therapy, such as the host immune response, the use of viral vectors, or mutagenesis [155].

CAR (chimeric antigen receptors)-T cell immunotherapy is also rapidly developing. CARs are synthetic receptors transferred to T cells of the patient to reprogram them and promote their attack to cancer cells. One of the main mechanisms of escape from the immune system in cancer cells is TGFβ secretion [156]. TGFβ not only suppresses the effector role of T cells, but also leads their differentiation to a regulatory phenotype. Treg cells can, in turn, produce more TGFβ and favor the tolerance to the tumor. With this in mind, TGFβ-responsive CAR-T cells have been engineered. These cells were able to improve the antitumor efficacy of neighboring cytotoxic T cells. Furthermore, TGFβ CAR-expressing Tregs do not exhibit TGFβ-induced immunosuppression, thus it is likely that this approach will be translated into clinical practice in a not too distant future [157].

What seems likely is that, whether through the therapeutic strategies included in this review or through new ones that will develop in the future, TGFβ will remain one of the key drug targets in cancer treatment.

## 6. Methods

### 6.1. Study Design

A systematic literature review was performed focusing on therapeutic strategies for cancer treatment that targeted TGFβ or any of the molecules involved in its synthesis, activation, or signaling.

### 6.2. Search Strategy

The search was conducted on MEDLINE database by using the free access search engine at the legacy PubMed site through the US National Library of Medicine portal (https://www.ncbi.nlm.nih.gov/pubmed/). Manuscripts were identified under the following search strategy: (transforming growth factor beta [Title/Abstract]) AND (cancer treatment [Title/Abstract] OR cancer therapy [Title/Abstract] OR ((therapeutic strategy [Title/Abstract] OR therapeutic target [Title/Abstract]) AND cancer [Title/Abstract]))).

Additionally, clinical trials were screened on the American and European official webpages www.clinicaltrials.gov and www.clinicaltrialsregister.eu through the simultaneous search of the terms “transforming growth factor” and “cancer” by means of the particle “AND”.

### 6.3. Exclusion/Inclusion Criteria

The results obtained from PubMed were filtered by article type, species, date of publication, and language. Original research articles were eligible for inclusion, with reviews being excluded from this analysis. Only those articles performed in humans and published in English during the five years previous to the search (from 1st October 2014 to 1st October 2019) were included. The results were managed with the reference management software Mendeley (Elsevier). Initially, the title and abstract of every article were revised to establish if they fulfilled a priori the inclusion criteria. Every original research article relating any of the main isoforms of TGFβ (TGFβ1, TGFβ2, and/or TGFβ3) with cancer were included, while articles focused on any other member of the TGFβ superfamily were excluded. The full-text of all potentially relevant articles were evaluated. Clinical trials were eligible for inclusion if normally concluded, and the recruitment status was “completed” during the period of our study (from 1st October 2014 to 1st October 2019). Results analyzing the success and possible side-effects of the therapeutic strategies proposed in the articles/clinical trials included were narratively presented and discussed.

## 7. Conclusions

Transforming growth factor (TGF)-β intervenes in critical processes linked to tumor development and progression that make this factor a promising molecular target in cancer treatment. The enormous variety of molecules involved in TGFβ synthesis, activation, or signal transduction, as well as those capable of indirectly, affect these mechanisms, represent potential therapeutic targets under research. The strategies assayed include antisense therapy for gene expression regulation, small molecule inhibitors or receptor traps to block the ligand-receptor interaction, as well as different compounds that avoid TGFβ activation or signaling. The combination of therapies aimed at different processes of carcinogenesis provides better results than a single treatment. Unfortunately, the translation into the clinics of these approaches requires a long way to go. The dual role of TGFβ in cancer, either as a tumor suppressor or as a tumor-promoter, together with the interindividual drug response variability, must be considered in cancer therapy. This makes necessary a previous thorough study of the cancer type and stage, together with the patient’s genetic predisposition, to ensure the effectiveness and safety of the treatment. Future studies will have to elucidate the success of new developing techniques in the fight against cancer.

## Figures and Tables

**Table 1:** Characteristics of the included studies proposing a miRNA-based therapeutic strategy.

miRNA	Target	miRNA Level in Pathological State	Cancer Type	Reference
miR-143-3p	Cystatin	Decreased	Ovarian cancer	Guan et al. [43]
miR-143	SMAD3	Decreased	NSCLC	Cheng et al. [45]
miR-124	SMAD4	Decreased	NSCLC	Zu et al. [46]
miR-155	TβRII	Increased	Gastric cancer	Qu et al. [47]
miR-155	SMAD2/3	Increased	Colon cancer	Velázquez et al. [48]
miR-155	SMAD2/3	Decreased	Prostate cancer	Ji et al. [49]
miR-520c	TβRII	Decreased	Glioma	Hu et al. [50]
miR-17-5p	TβRII	Increased	Gastric cancer	Qu et al. [51]
miR-323-3p	SMAD2/3	Decreased	PDAC	Wang et al. [20]
miR-367	SMAD7	Increased	PDAC	Zhu et al. [52]
miR-106b	SMAD7	Increased	Gastric cancer	Yu et al. [53]
miR-455-3p	p-SMAD2	Increased	ESCC	Liu et al. [54]
miR-592	TGFβ2	Decreased	Breast cancer	Hou et al. [55]
miR-153	TGFβ2, p-SMAD2/3	Decreased	Osteosarcoma	Niu et al. [56]
miR-16	TGFβ	Decreased	Glioma	Wang et al. [57]
miR-3591-5p	TGFβ, SMAD2/3	Increased	Lung cancer	Lu et al. [58]
miR-27a	SMAD2/4	Increased	Lung cancer	Chae et al. [59]

NSCLC, non-small cell lung cancer; PDAC, pancreatic ductal adenocarcinoma; ESCC, esophageal squamous cell carcinoma.

## Data Availability

Not applicable.
